# Functional MR elastography measures visual cortex stiffening proportional to visual contrast intensity in regions of activation

**DOI:** 10.1162/imag_a_00172

**Published:** 2024-05-08

**Authors:** Harish R. Palnitkar, Matthew C. Murphy, Yi Sui, Kevin J. Glaser, Armando Manduca, Kirk M. Welker, Norbert Campeau, John Huston, Richard L. Ehman, Arvin Arani

**Affiliations:** Department of Radiology, Mayo Clinic, Rochester, MN, United States; Department of Physiology and Biomedical Engineering, Mayo Clinic, Rochester, MN, United States

**Keywords:** functional MR elastography (fMRE), fMRI, visual stimulus, luminance-matched contrast intensity

## Abstract

Functional MRI (fMRI) is widely used to spatially localize neural activity in the brain associated with functional stimuli. Functional MR Elastography (fMRE) has recently been introduced as a complementary approach that measures the mechanical response to functional stimulus. The hypothesis of the current study is that the stiffness change in fMRE is proportional to the underlying neural activity. This hypothesis is tested by measuring the median stiffness change in the visual cortex as a function of luminance-matched contrast intensity of a checkerboard visual stimulus in 16 healthy subjects. The fMRE signal in the visual cortex was observed to be proportional to the contrast intensity of the visual stimulus. In regions of activation, fMRE signal increased in the range of 2 ± 1% to 5.8 ± 1% and fMRI signal increased by the expected 0.4 ± 0.2% to 0.9 ± 0.2%, for contrast levels of 5% to 100%, respectively. In conclusion, this study shows that the fMRE signal in the visual cortex can be directly modulated by the contrast intensity of a visual stimulus. The presence of some overlap between fMRI and fMRE regions of activation may suggest two distinct mechanisms governing the fMRI and fMRE signals, which will be investigated in future studies.

## 1 Introduction

Functional MRI (fMRI) using contrast produced by hemodynamic effects is long established as a sensitive method for mapping neural activity in the brain (Ogawa et al., 1990,^1992^). Since fMRI can be performed noninvasively, it has been a valuable tool for localizing task-related activity (Lingnau et al., 2009;Meier et al., 2008;Pugh et al., 1996), for exploring the network architecture of the brain (Kajimura et al., 2023;Vidaurre et al., 2021), and for presurgical planning with tumors (Dimou et al., 2013) and patients with epilepsy (Benjamin et al., 2018). While fMRI has the advantages of noninvasiveness and whole-brain coverage, the spatial and temporal resolution of the method are intermediate compared to other techniques (Mantini et al., 2010) such as Electroencephalography (EEG) and Magnetoencephalography (MEG), which limits the inferences that can be drawn from these data. For this reason, additional modes of interrogation may prove valuable for mapping neural activity with accuracy in time and space.

Recent studies have explored the potential that neural activity may be associated with changes in brain mechanical properties, perhaps due either to a change in blood volume that alters the composite behavior of the material, or due to other processes such as cellular swelling (Parker, 2017a,2017b;Patz et al., 2019). In support of this hypothesis, it has been shown that brain stiffness is modulated by neural activity using a functional magnetic resonance elastography (fMRE) approach (Forouhandehpour et al., 2021;Lan et al., 2020;Patz et al., 2019). Magnetic Resonance Elastography (MRE) is an MRI-based method for noninvasively measuring tissue mechanical properties using three steps: (1) introduction of shear waves into the tissue of interest via an external vibration; (2) imaging the resulting displacement field by phase-contrast MRI; and (3) mathematical inversion of the displacement field to estimate the underlying mechanical properties (Muthupillai et al., 1995;Romano et al., 1998,^2000^). While various investigators have demonstrated the feasibility of fMRE as a novel biomarker to detect neuronal activation, the underlying mechanism of stiffness change remains to be identified. The latter is not the focus of the current study. To date, a consensus has not been reached regarding the direction of stiffness change with respect to neural activity: the sign of observed stiffness change has been linked to the duration of functional stimulus (Table 2). The relationship between the sign of stiffness change and the time scale of the stimulus is still under investigation.

This study aimed to better understand the brain mechanical response to visual stimulation intensity, with the hypothesis that stiffness changes are proportional to underlying neural activity. This hypothesis was tested by measuring the magnitude of stiffness change in the visual cortex as a function of stimulus contrast intensity, relying on previous BOLD fMRI studies that demonstrated stimulus contrast is proportional to multi-unit activity and local field potential power (Boynton et al., 1999;Henrie & Shapley, 2005;Logothetis et al., 2001).

## 2 Materials and Method

### 2.1 Study population

This study was reviewed and approved by our Institutional Review Board, and participants provided written informed consent prior to being enrolled. Sixteen subjects (8 male and 8 female, ages in range 20–60, mean age = 40) were recruited. To ensure familiarity with the experiment, prior to entering the scan room, each subject was trained on all the functional paradigms and contrast intensities performed during the functional MRI experiment using the PRISM Acquire Software (Prism Clinical Imaging, Elm Grove, WI).

### 2.2 Design of experiment: Block paradigm

**Fig. 1.**
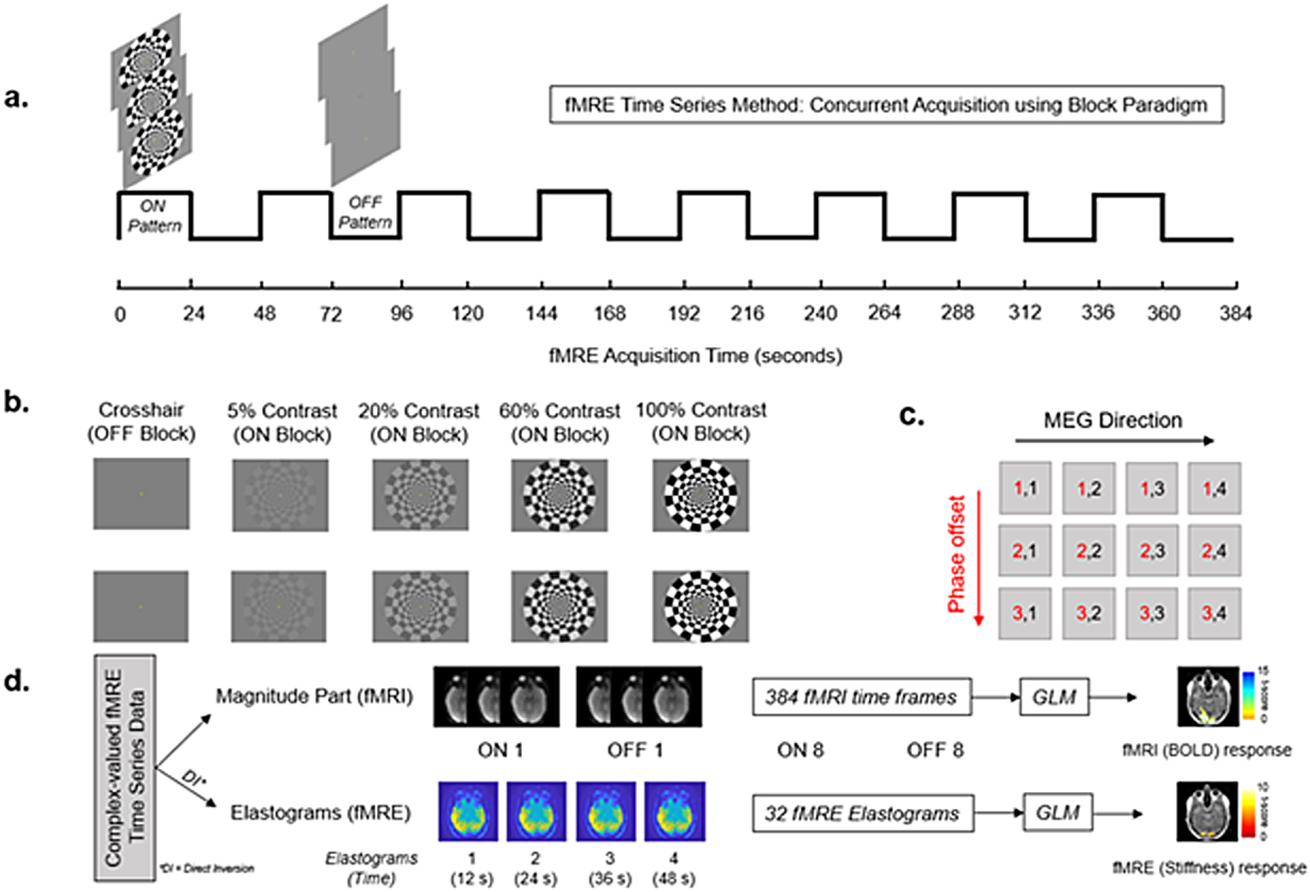
Concurrent fMRI-fMRE Acquisition: (a) Block paradigm (time series method); (b) 4 paradigms of 5%, 20%, 60%, and 100% contrast intensity were shown to each participant with contrast reversal every 0.1 s; (c) MRE acquisition scheme using 4 Motion Encoding Gradients (MEGs) and 3 phase offsets; (d) Data analysis using General Linear Model (GLM).

The current work used the concurrent block paradigm approach proposed byLan et al. (2020)with a block duration of 24 s and 16 blocks as shown inFigure 1a. The concurrent fMRI-fMRE approach pioneered byLan et al. (2020)allows simultaneous acquisition of both fMRI (BOLD) and fMRE (Elastograms) from the same complex valued data. The ON blocks were luminance-matched 5%, 20%, 60%, and 100% visual contrasts with a 10 Hz frequency of flickering (Fig. 1b). A red dot was displayed at the center of the screen at equal number of times during both ON and OFF blocks. To maintain attention, participants were asked to count the number of times they saw the red dot during an experiment. The subjects were usually able to accurately count the number of times they saw the red dot during each scan; if the participants counted the wrong number of dots, the scan was repeated. The number of times a red dot appeared during ON and OFF blocks was kept constant to ensure consistency in the experiment. In addition, we had performed dry runs both with red dot and without red dot on same subject during the same study. We found no effect of presence of red dot on the region of activation for both fMRI and fMRE. Thus, counting red dots served as a way to keep the participant active during the scan without leading to a confounding effect on region of activation.

### 2.3 Concurrent fMRI-fMRE data acquisition

Subjects were scanned on a high-performance compact 3T MR system (GE Global Research, Niskayuna, NY) in the supine position (Foo et al., 2018). During the examination, a 3D MPRAGE T1 anatomical scan (GRE, FOV = 25.6 cm x 25.6 cm x 20 cm, voxel size = 1 x 1 x 1.2 mm, TR/TE = 2200/2.5 ms) was performed to localize the visual cortex for functional imaging. This was followed by 4 fMRE scans with varying intensities of visual contrast. To limit bias, the order of the scans was randomized for each individual subject. The fMRE experiments used a bandwidth = ±250 kHz, and 0th- and 1st-order moment nulled motion encoding gradient lobe on each side of the refocussing radiofrequency pulse synchronized to the mechanical motion. Hadamard motion encoding was used with 4 MEG directions (Guenthner et al., 2017) and 3 phase offsets sampled over one period of the vibrational motion (16.67 ms). In-plane acceleration was used. Multi-band excitation pulses were not used. We used an MEG frequency of 60 Hz. The GE Compact 3T MRI scanner used in this study had a gradient amplitude of 80 mT/m and a gradient slew rate of 700 T/m/s. Mechanical vibrations were introduced to the brain through a soft pillow-like passive driver as previously reported (Arani et al., 2018;Murphy et al., 2011). The volume of image acquisition for the fMRE scans was selected in such a manner that the 12 slices spanned each participant’s primary visual cortex (V1) with the middle slice centered on the calcarine sulcus. After 3 s of discarded acquisition, to allow for gradual ramping of the vibrations and to achieve steady-state motion, the block paradigm on Prism acquire software (Prism Clinical Imaging, Elm Grove, WI, USA) was manually triggered from a separate computer connected to an LED display monitor (NordicNeuroLab, Bergen, Norway) inside the MRI room. Our MRE sequence required 12 s to generate a single volume (12 slices in 22.4 cm FOV). Therefore, our functional block paradigm consisted of 16 blocks (8 ON + 8 OFF) with a block duration of 24 s per block. This resulted in a scan time of 384 s per scan, with a separate fMRE scan for each of the 4 contrast intensities (5%, 20%, 60%, and 100%). More temporal resolution is necessary to determine if habituation to the visual pattern could potentially affect the fMRE response.

Two gradient echo (EPI-GRE) fMRI scans were performed towards the end of the experiment in order to compare our SE-fMRI (magnitude of the MRE data) and fMRE (elastogram) activation maps against a standard clinical EPI-GRE-fMRI acquisition. The EPI-GRE-fMRI scans were limited to 2 visual contrast intensities (5% and 100%) to limit total exam time.Table 1lists the scan parameters used in both the studies.

**Table 1.** fMRE and EPI-GRE-fMRI experiment parameters.

Parameter	fMRE acquisition	EPI-gradient echo clinical fMRI
Pulse sequence	SS SE EPI MRE (4 MEG, 3 phase offset)	Gradient Echo
Mechanical frequency (MRE)	60 Hz	NA
TR/TE	1000 ms / 64.5 ms	2000 ms / 28 ms
No. of slices	12	50
Slice thickness, gap	3.5 mm, 0 mm	3 mm, 0 mm
FOV	22.4 cm x 22.4 cm	24 cm x 24 cm
Voxel size	3.5 mm x 3.5 mm x 3.5 mm	3.75 mm x 3.75 mm x 3 mm
Matrix size	64 x 64 (interpolated to 128 x 128)	64 x 64 (interpolated to 128 x 128)

### 2.4 fMRI pre-processing and data analysis

**Fig. 2.**
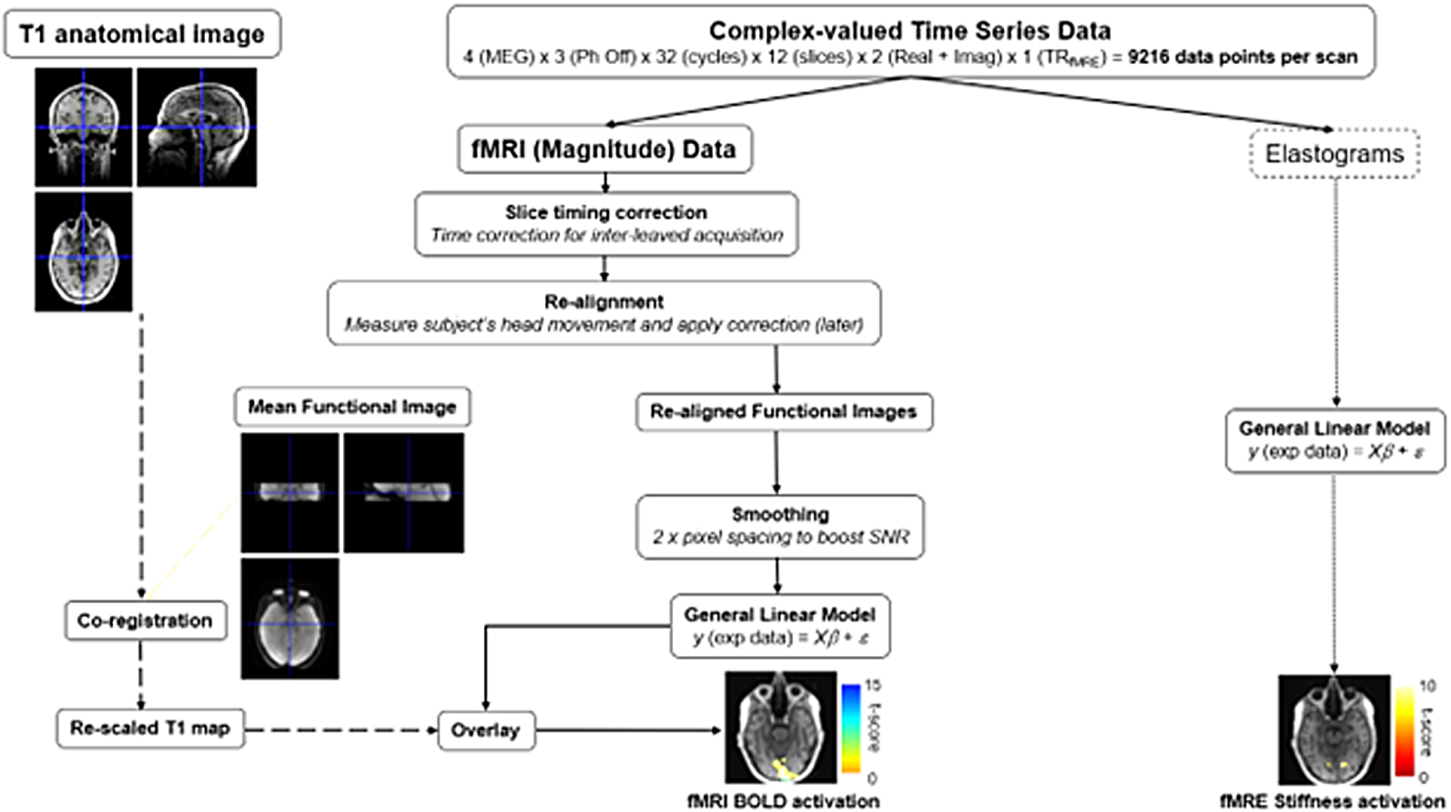
fMRI-fMRE data analysis pipelines for fMRE stiffness and fMRI BOLD processing from the acquired complex-valued MRE data set.

Figure 2schematically depicts the data analysis pipelines for both SE-fMRI (magnitude) and fMRE (elastograms). For the magnitude component of the complex valued MRE data, we used a TR_fMRI_= 1 s, leading to 384 SE-fMRI timeframes for the whole MRE acquisition. Statistical Parametric Mapping (SPM12, UCL, London, UK) was used to analyze the data. During pre-processing, slice timing correction, re-alignment, normalization, co-registration with anatomical images, and smoothing were applied to the data. The task regressor was the block design convolved with the canonical Hemodynamic Response Function (HRF) (Chen & Glover, 2015). Time and dispersion derivatives for the regressor function and motion correction were used. In order to determine regions of activation, a significance threshold of uncorrected p < 0.001 was used. We computed the number of active voxels and the percent signal change within active voxels for each case.

### 2.5 fMRE (elastograms) preprocessing and data analysis

A time-series of elastograms was computed from each 12-s bin of MRE stiffness data. Interslice phase discontinuities were reduced by using a previously described in-plane, high-pass filter (Murphy et al., 2012) and wave images were unwrapped using a graph cut method (Bioucas-Dias & Valadao, 2007). The curl of the displacement field was computed to remove the effects of longitudinal waves, and the temporal Fast Fourier Transformation (standard FFT applied along time dimension) was used to isolate wave information at 60 Hz. Smoothing of first-harmonic curl images was performed using a 7 x 7 x 5 quartic smoothing kernel (Romano et al., 2000), and shear stiffness was computed by direct inversion (Oliphant et al., 2001).

As above, the time series of elastograms was fit by a general linear model in SPM12. To approximate the optimized Gamma response function reported byLan et al. (2020), the standard HRF was modified to change the time of peak response from 6 s (default) to 8 s and lengthen the time from peak to undershoot from the default 6 s to 100 s (Lan et al., 2020). This modified HRF was then convolved with the block paradigm to create the design matrix for the General Linear Model (GLM).

### 2.6 Statistical analysis

The results of the GLM were thresholded at p < 0.001, and SPM was used to compute the number of activated voxels and the percentage change in the signal due to activation. Bootstrapping analysis (Freedman, 1981) was done for n = 16 sample points (equal to the number of participants in the cohort), by resampling the data to n* = 10,000 points. Note that separate bootstrapping analysis was done for data corresponding to each of the 6 plots shown inFig. 4(a-f), for each of the 4 contrasts. This was followed by a Linear Mixed-Effects Model analysis with fixed effects for contrast intensity on all 4 contrasts for the 16 volunteers.

## 3 Results

### 3.1 Effect of increasing luminance-matched contrast intensity on stiffness of visual cortex

**Fig. 3.**
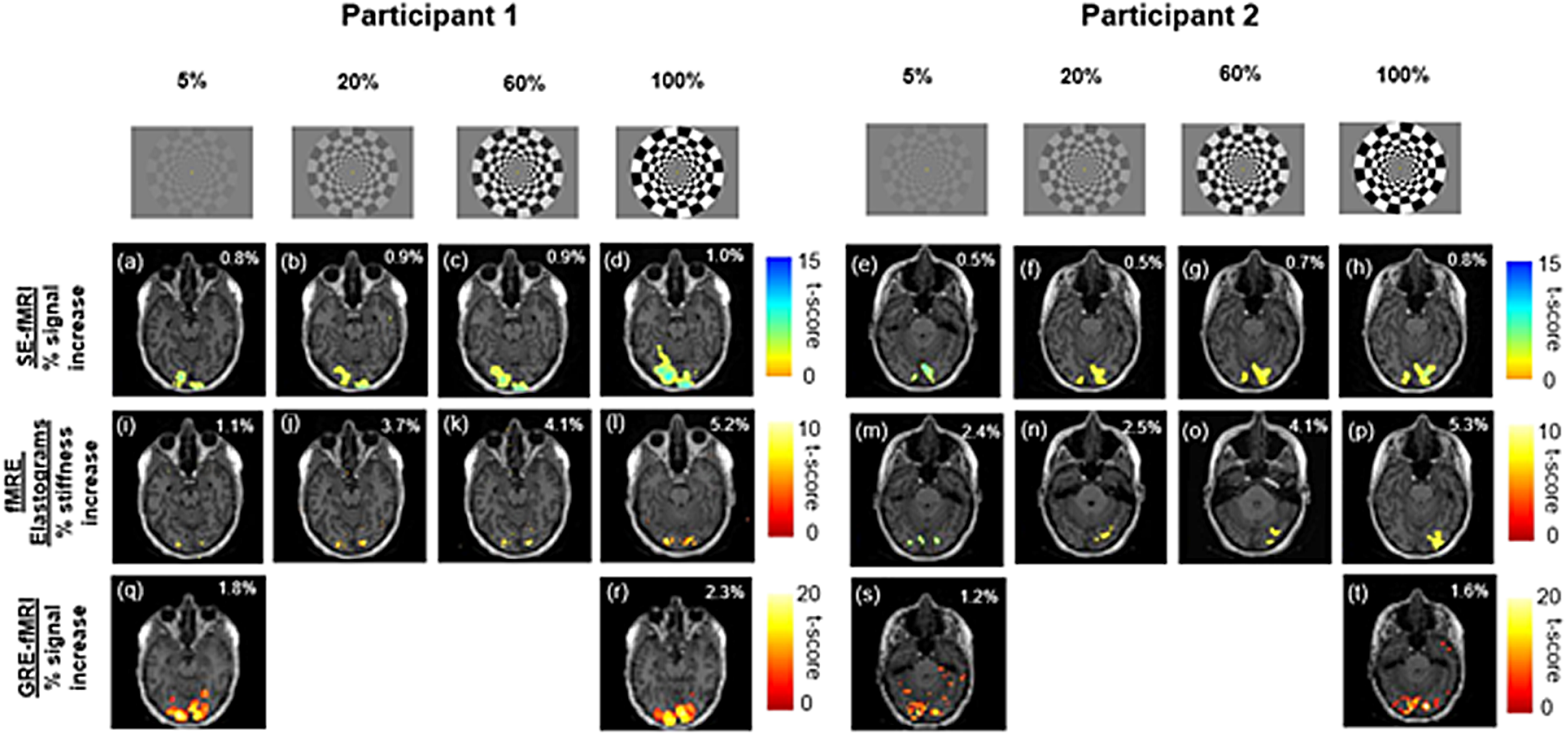
A comparison of regions of activation (p < 0.001, sample size = 16 participants), for 2 participants:**first row**shows the visual patterns with increasing contrast intensity (5%, 20%, 60%, and 100%);**second row: (a)-(d)**and**(e)-(h)**are activation maps from SE-fMRI (from fMRE magnitude data) for participants 1 and 2 respectively;**third row: (i)-(l)**and**(m)-(p)**are activation maps from fMRE elastograms for participants 1 and 2 respectively;**fourth row: (q)-(r), (s)-(t)**are activation maps from conventional EPI-GRE-fMRI. The percentage values (inset of each activation map) indicate percent signal increase due to visual stimulation.

Activation maps for two participants for SE-fMRI from fMRE magnitude data, fMRE elastograms, and EPI-GRE-fMRI are shown inFigure 3rows 2, 3, and 4 respectively. Activation was in the visual cortex region. An increase in the visual contrast intensity led to a linearly proportional increase in signal across all the participants for SE-fMRI, fMRE elastograms, and EPI-GRE-fMRI. A closer look at the fMRE elastograms (Fig. 3(i-l) and (m-p)) shows that there is some overlap between the SE-fMRI and fMRE elastogram regions of activation. In addition, the response to the highest visual contrast is highest for fMRE elastograms (5.8 ± 1%, Cohen’s d = 5.8) when compared to SE-fMRI (0.9 ± 0.2%, Cohen’s d = 1.0) and EPI-GRE-fMRI (1.7 ± 0.5%).

Our observations are consistent with the findings reported byLan et al. (2020).

**Fig. 4.**
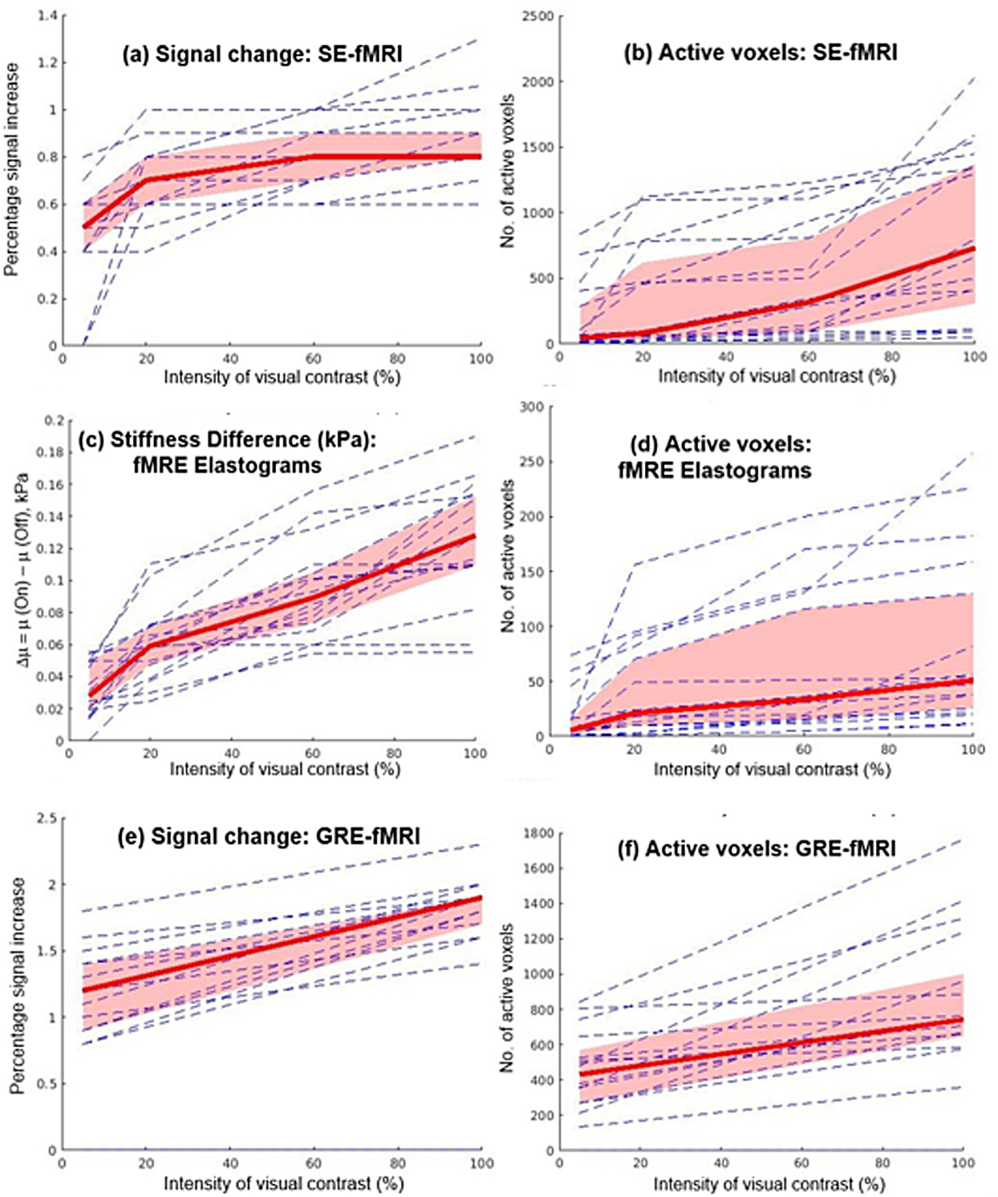
Plots of individual and median values of signal change and number of activated voxels for: (a, b) SE-EPI fMRI, (c, d) fMRE Elastograms, and (e, f) EPI-GRE-fMRI. Median value (bold red line) and 95% Confidence Interval (in red) were obtained from Bootstrap analysis of n = 16 sample points, re-sampled to n’ = 10,000 sample points.

Figure 4shows plots of increase in the percentage signal due to an increase in the contrast intensity of the visual stimulus, along with a plot of the number of active voxels, for SE-fMRI, for fMRE Elastograms, and for EPI-GRE-fMRI.Figure 4also shows the median and 95% CI values as a function of the contrast intensity. It is observed that both signal intensity (Fig. 4(a), (e)) and stiffness (Fig. 4(c)) increase proportionally with the contrast intensity of the visual stimulus. We did a linear regression analysis in the contrast range from 5% to 100% and found the following relationship between ∆µ and percent contrast intensity:

Δµ=0.0013(% contrast intensity)+0.03, kPa (1)

RMS error of the linear fit (eq. 1) was found to be 0.03 kPa. A linear mixed-effect analysis with fixed effect for contrast intensity led to an estimated p < 0.0001, indicating a significant correlation between the intensity of visual contrast and signal change.

Similarly, we also performed a linear regression analysis in the contrast range from 5% to 100%, and we found the following relationship between percentage signal increase (SE-fMRI) and percent contrast intensity:

$$\begin{array}{l} \text{Percent signal increase }\left(\text{SE}-\text{fMRI}\right)=0.15\\ \;\;\;\times \left(\text{\% contrast intensity}\right)+0.7 \end{array}$$ (2)

The RMS error of the linear fit ineq. (2)was found to be 0.7. A linear mixed-effect analysis with fixed effect for contrast intensity gave an estimated p < 0.0001, which indicates a significant correlation between intensity of visual contrast and percentage signal increase (SE-fMRI).

We also performed control scan (dry run) on a single volunteer where there was no visual stimulus (fMRI-fMRE block paradigm where both ON and OFF blocks displayed blank screen). The results of this dry run showed no activation in both fMRI and fMRE scans. This confirms that the results we observe in our fMRI-fMRE data are indeed from the visual stimulus.

## 4 Discussion

The results of this study demonstrate that MRE-assessed stiffness changes in the visual cortex in response to visual stimulation are affected by stimulus intensity. We found that stiffness, measured with MRE, increases linearly as a function of percent contrast intensity (eq. 1), where at 100% contrast, we found ∆µ = 0.16 ± 0.03 kPa. Maps of temporal SNR and stiffness (fMRE) are provided inSupplementary Figures 2 and 3respectively. We also note that there is a noticeable overlap of regions of activation for SE-fMRI and fMRE stiffness maps at our significance threshold of p < 0.001 (Fig. 3). This may suggest two distinct mechanisms that govern stiffness response and BOLD response to a visual stimulus. Future studies will explore the relationship between stiffness change and BOLD response, by using a shorter block duration to de-couple faster stiffness responses from the slower hemodynamic responses occurring in the 8-11 s time frame. We also observed that the number of active voxels was highest for GRE-fMRI and lowest for fMRE elastograms, suggesting that our elastogram data is at the lower limit of the SNR needed to detect this mechanical signal.

We further note fromFigure 4(Lan et al., 2020) that though the regions of fMRI and fMRE activation are within the primary visual cortex region (V1), those regions are not exactly the same (Lan et al., 2020;Resultssection, paragraph no. 3). These findings are consistent with the findings reported in our study (Supplementary Table 1) in which we observe activation of regions inside the primary visual cortex (V1) for both fMRI and fMRE; these regions do not exactly overlap with each other. This observation from both our study andLan et al. (2020)may point towards the possibility of two distinct mechanisms governing neurovascular (fMRI) and neuromechanical (fMRE) activation.

The fMRE field is in its early stages and therefore a summary of the work that has been published is listed inTable 2. The authors note with caution that the mechanical frequency of excitation (driver frequency), the nature of the functional stimulus, the frequency of the stimulus, and the block duration influence the computed stiffness. This points towards diverse functional mechanisms that contribute to the differences reported in stiffness in brain tissue. As a result, a consensus on the impact of stiffness changes resulting from functional stimuli has not been established. Some studies have reported an increase in stiffness while others have reported a reduction in stiffness response (Table 2). The underlying reason for stiffness change is still an active area of research and has not been established. Protocols that use longer block durations (24 s) have reported an increase in stiffness of activated regions (for both visual and motor cortex), while protocols that use shorter block durations (<1 s) have reported a reduction in the stiffness of the activated regions. In our preliminary work on the motor cortex (using long block durations of 24 s), we have observed an increase of stiffness of activated regions in the motor cortex.

**Table 2.** Summary of prior work aimed at understanding the reasons for stiffness change due to functional activation.

Year/Author	Functional stimulus	fMRE parameters	Observation
2014 Fehlner et al. (2014)	Visual checkerboard	• MRE driver frequency = 25, 30, 40, 50 Hz; • Visual stimulus frequency = 8 Hz; • Block durations = 8 s, 36 s	2.5% reduction in whole brain viscoelasticity
2015 Holub et al. (2015)	Motor cortex stimulus (Finger tapping)	• MRE driver frequency = 30 Hz; • Block duration = 6 min OFF, 6 min ON (just once); • Fingertapping: All right-hand fingers	Softening (ROI) by 30%
2015 Patz et al. (2015)	Auditory stimulus (Mouse model)	• MRE driver frequency = 1 kHz; • No block paradigm used; • Auditory stimulus was 1 kHz sound from the driver and the gradients	Stiffness increase by 50%
2017, 2018 Patz et al. (2017 , 2018 , 2019 )	Murine hind limb stimulation	• MRE driver frequency = 1 kHz; • Block duration = 9 s (slow), 0.9 s (fast), 100 ms (ultrafast); • Hind limb stimulation using electric pulse	Decrease in stiffness by 14%
2018 de Arcos et al. (2018)	Human visual fMRE	• MRE driver frequency = 50 Hz; • Visual stimulus switching at 1.35 Hz; • Visual stimulus = Monocular flashing light from a fiber optic source	Increase in stiffness by 10% (consistent with findings of our work)
2020 Lan et al. (2020)	Contrast Reversing Visual checkerboard	• MRE driver frequency = 60 Hz; • Block duration = 18 s, 24 s, 36 s	Increase in stiffness by 6–11% (consistent with our work)
2021 Forouhandehpour et al. (2021)	Contrast Reversing Visual checkerboard	• Intrinsic MRE: pulse at 1 Hz; • Block duration: 300 s • Contrast reversing frequency = 1/8 s	Both increase and decrease in stiffness of activated voxels inside visual cortex
2022 Mishra et al. (2022)	Motor cortex stimulus (finger tapping)	• MRE driver frequency = 50 Hz • Block duration = 2 s	Reduction in stiffness by 30%
2023 Palnitkar et al. (2023)	Contrast reversing visual checkerboard	• MRE driver frequency = 60 Hz; • Block duration = 24 s; • Investigated the effect of increasing contrast intensity and frequency of flickering of visual stimulus on fMRI-fMRE response	5–6% increase in the stiffness.Both fMRI and fMRE are modulated by increasing contrast intensity and frequency of visual stimulus.

From the above investigations, we note that some researchers have demonstrated a reduction in the stiffness of activated voxels, while others have shown an increase in the stiffness. We believe that these observations are impacted by variations in fMRE experimental parameters, including: the type of functional stimulus (visual or motor or auditory); the block duration (long duration such as 300 s (Forouhandehpour et al., 2021) or ultra-short duration such as 100 ms (Patz et al., 2019)); the MRE driver frequency; and participant attentiveness. The current work is a step towards understanding the relationship between stiffness changes with respect to contrast intensity.

Another important finding is that regardless of the technique used, each study demonstrates that brain tissue stiffness changes with a functional stimulus. The acute nature of these stiffness changes and the small amplitudes of these stresses support the idea that the brain may be in a non-linear elastic regime under homeostasis. This is important because MRE may be sensitive to a potential link between pressure changes and brain stiffness. This hypothesis is supported by prior work in a swine model (Arani, A. et al., 2018) which established a relationship between MRE-measured brain stiffness and elevated intracranial pressure (ICP). Following surgical release of CSF at the beginning of this experimental study, stiffness decreased and then stayed stable until baseline CSF pressures were reached (~25 mmHg). Once the pressure increased beyond baseline values, stiffness increased with pressure. In our current study, a controlled increase in the visual stimulus led to small incremental displacements in the brain parenchyma. These small displacements then led to measurable increase in the stiffness of activated regions in the brain, again suggesting a non-linear elastic behavior and a direct relationship between stiffness and pressure changes in healthy brain tissue. Understanding the true mechanism behind the cause of this stiffness change is outside of the scope of this paper and will need to be explored in future studies.

Due to the exploratory nature of this study, there were some limitations. The number of participants in this study was limited to 16; in the future, this pool can be expanded to include more participants. In addition, the activation maps are impacted by participant attentiveness. Some subjects experienced difficulty in remaining engaged during the entire scan even after using the red dot technique as described inSection 2.2. In the future, a superior technique to keep a track of participant attentiveness would be the use of eye tracking as described inFrey et al. (2021)andKanowski et al. (2007). Another limitation of our study is the low temporal resolution of the fMRE data, leading to reduced statistical power compared to a standard EPI-GRE fMRI experiment. In the future, we hope to use custom sequences with more ON/OFF blocks to add statistical power to our GLM.

The fMRE signal response is affected not only by participant attentiveness, but also by experimental parameters such as type of stimulus, block duration (fast vs. slow stimulus), flickering frequency of visual checkerboard, and the MRE driver frequency. We believe that to enable a comparison between the results obtained by various research groups, and to enable clinical application of fMRE, a standardized acquisition protocol is necessary. Future work will be aimed at increasing the temporal SNR in fMRE, and at exploring the relationship between the mechanisms governing fMRI and fMRE.

## 5 Conclusion

In conclusion, the current work characterizes the functional MRE signal in the visual cortex in response to visual stimulus. Stiffness change, measured with fMRE, increases as a function of the luminance-matched contrast intensity of a visual stimulus. Future studies are needed to understand the relationship between fMRE and fMRI signal responses, with a particular focus on improving the temporal SNR of the fMRE signal.

## Supplementary Material

Supplementary Material

## Data Availability

Raw data from fMRI-fMRE scans and MATLAB code are available upon request.
