# Supplementary material for: Functional MR elastography measures visual cortex stiffening proportional to visual contrast intensity in regions of activation
Source: Imaging Neurosci (Camb). 2024 May 8;2:imag-2-00172. doi: 10.1162/imag_a_00172 (PMC12031641; doi:10.1162/imag_a_00172)

Supplementary Figure 1

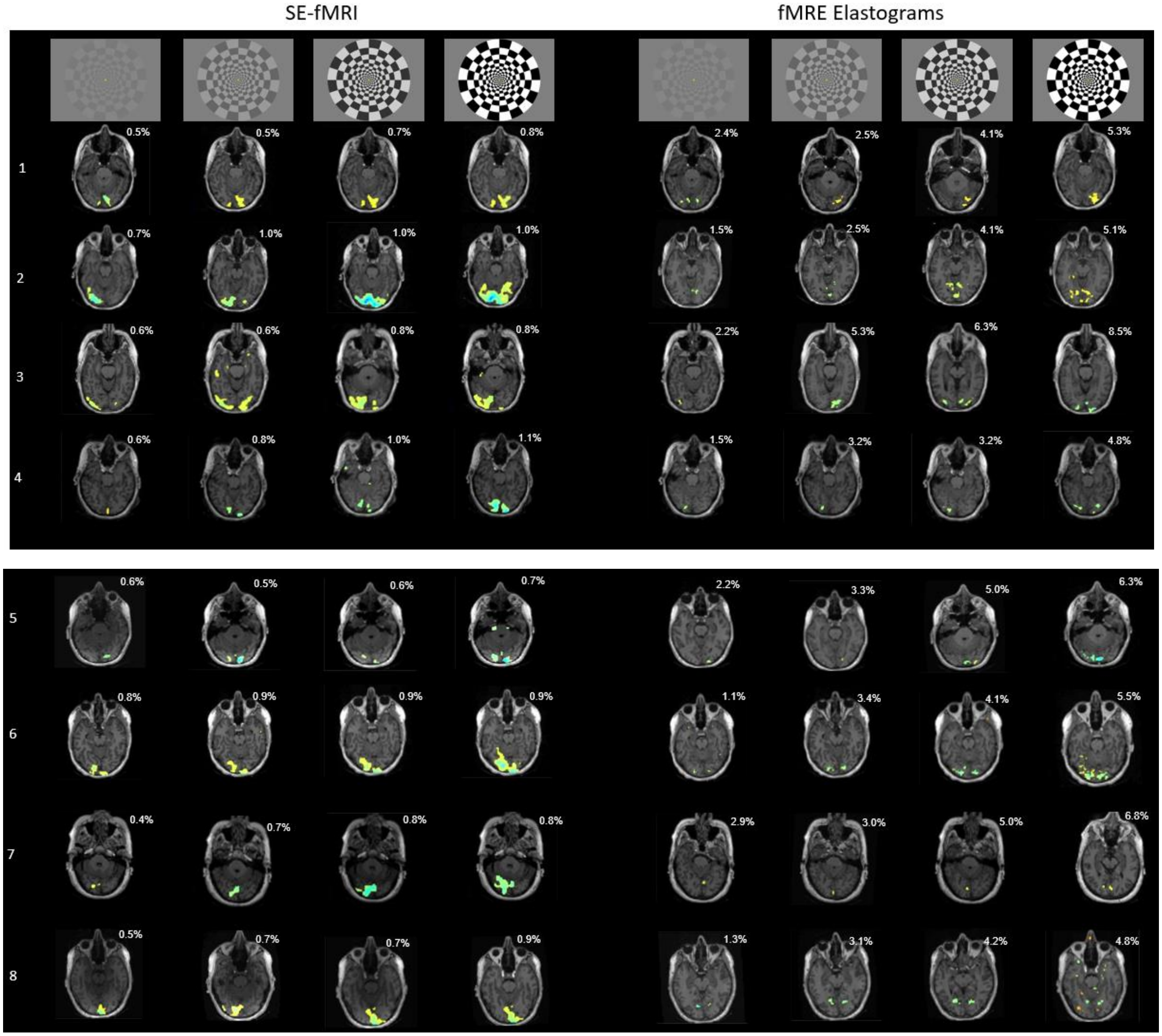

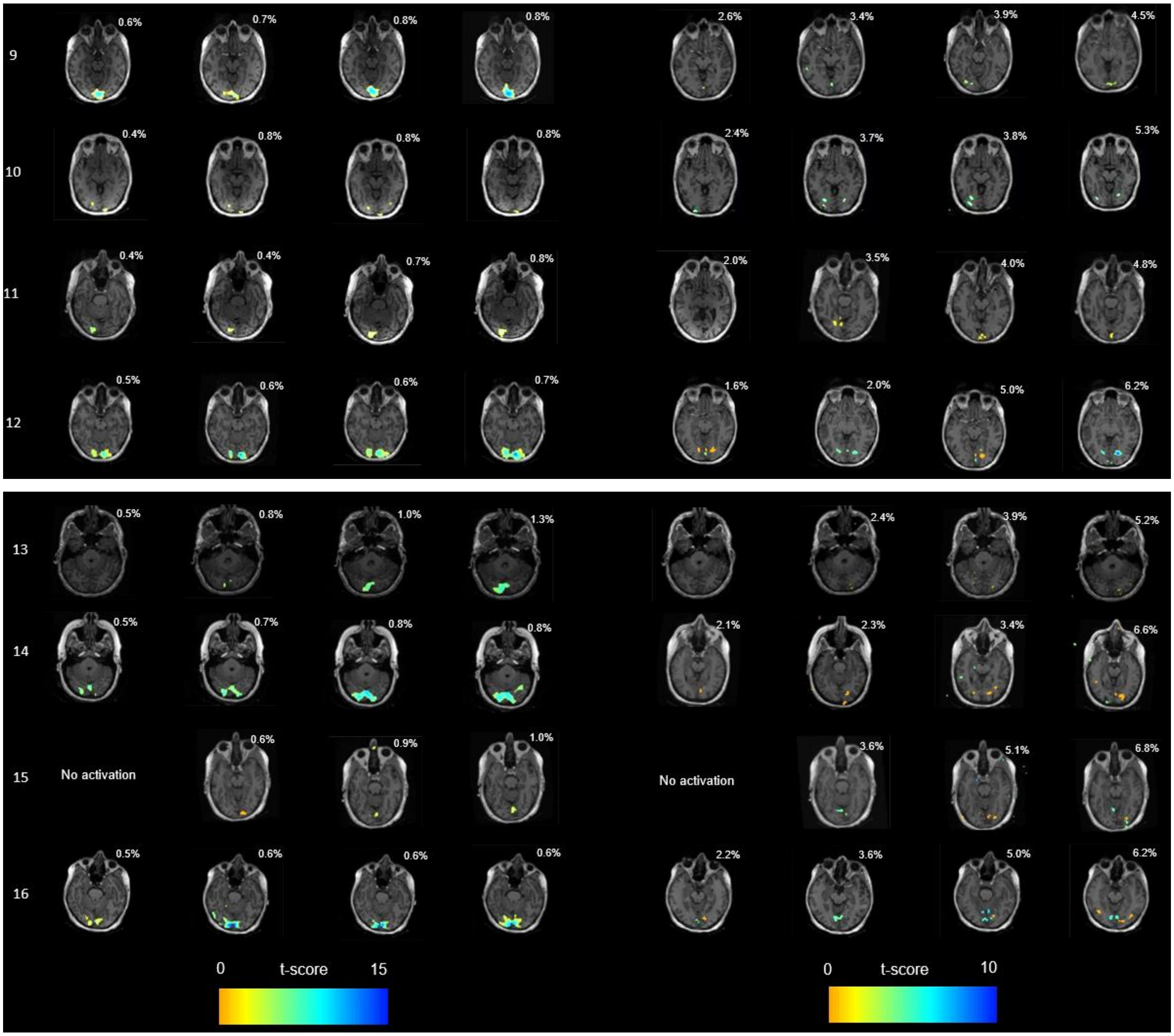

Legend: The percentage values correspond to % signal change (BOLD fMRI) for the images in columns 1 to 4 and the percentage values correspond to % stiffness increase (fMRE) for the images in columns 5 to 8.

Supplementary Table 1: List of number of active fMRI and fMRE voxels inside V1

Participant No.	Volume of V1 (mm^3)	fMRI (Total no. of active voxels inside V1) Visual contrast intensity				fMRE (Total no. of active voxels inside V1) Visual contrast intensity			
		5%	20%	60%	100%	5%	20%	60%	100%
**1**	32516.4	50	56	61	111	6	10	15	38
**2**	41109.6	209	275	369	771	60	82	170	182
**3**	34511.4	117	224	300	325	15	78	110	160
**4**	35515.2	15	67	86	196	2	20	30	70
**5**	27077.4	35	64	104	180	5	13	24	50
**6**	34398	87	157	271	333	10	20	40	60
**7**	26308.8	14	25	89	93	1	2	8	12
**8**	34465.2	17	32	145	240	4	8	42	180
**9**	22134	32	40	117	138	5	15	46	58
**10**	25200	21	22	29	44	5	10	14	24
**11**	28497	70	95	345	405	1	1	5	11
**12**	23541	40	60	100	140	8	28	50	60
**13**	26040	0	20	60	100	0	3	15	30
**14**	18828.6	10	46	93	129	1	5	14	26
**15**	22692.6	0	8	39	94	0	2	12	40
**16**	22024.8	31	78	100	200	5	20	40	104

Supplementary Table 2: Percentage of V1 with active voxels in relation to volume of V1

Participant No.	Volume of V1 (mm^3)	fMRI (Percentage of V1 with active voxels in relation to volume) Visual contrast intensity				fMRE (Percentage of V1 with active voxels in relation to volume) Visual contrast intensity			
		5%	20%	60%	100%	5%	20%	60%	100%
1	32516.4	6.6	7.4	8.0	14.6	0.8	1.3	2.0	5.0
2	41109.6	21.8	28.7	38.5	80.4	6.3	8.6	17.7	19.0
3	34511.4	14.5	27.8	37.3	40.4	1.9	9.7	13.7	19.9
4	35515.2	1.8	8.1	10.4	23.7	0.2	2.4	3.6	8.5
5	27077.4	5.5	10.1	16.5	28.5	0.8	2.1	3.8	7.9
6	34398	10.8	19.6	33.8	41.5	1.2	2.5	5.0	7.5
7	26308.8	2.3	4.1	14.5	15.2	0.2	0.3	1.3	2.0
8	34465.2	2.1	4.0	18.0	29.9	0.5	1.0	5.2	22.4

9	22134	6.2	7.7	22.7	26.7	1.0	2.9	8.9	11.2
10	25200	3.6	3.7	4.9	7.5	0.9	1.7	2.4	4.1
11	28497	10.5	14.3	51.9	60.9	0.2	0.2	0.8	1.7
12	23541	7.3	10.9	18.2	25.5	1.5	5.1	9.1	10.9
13	26040	0.0	3.3	9.9	16.5	0.0	0.5	2.5	4.9
14	18828.6	2.3	10.5	21.2	29.4	0.2	1.1	3.2	5.9
15	22692.6	0.0	1.5	7.4	17.8	0.0	0.4	2.3	7.6
16	22024.8	6.0	15.2	19.5	38.9	1.0	3.9	7.8	20.2

Supplementary Table 3: Percentage of overlapping fMRI-fMRE regions in relation to total volume of V1

**Participant No.**	**Volume of V1 (mm^3)**	**Percentage of overlapping fMRI-fMRE region inside V1**			
		**Visual contrast intensity**			
		**5%**	**20%**	**60%**	**100%**
1	32516.4	12.0	17.9	24.6	34.2
2	41109.6	28.7	29.8	46.1	23.6
3	34511.4	12.8	34.8	36.7	49.2
4	35515.2	13.3	29.9	34.9	35.7
5	27077.4	14.3	20.3	23.1	27.8
6	34398	11.5	12.7	14.8	18.0
7	26308.8	7.1	8.0	9.0	12.9
8	34465.2	23.5	25.0	29.0	75.0
9	22134	15.6	37.5	39.3	42.0
10	25200	23.8	45.5	48.3	54.5
11	28497	1.4	1.1	1.4	2.7
12	23541	20.0	46.7	50.0	42.9
13	26040	0.0	15.0	25.0	30.0
14	18828.6	10.0	10.9	15.1	20.2
15	22692.6	0.0	25.0	30.8	42.6
16	22024.8	16.1	25.6	40.0	52.0

Supplementary Figure 2

SE EPI fMRE (Elastograms): Temporal SNR (Slices 1 to 12)

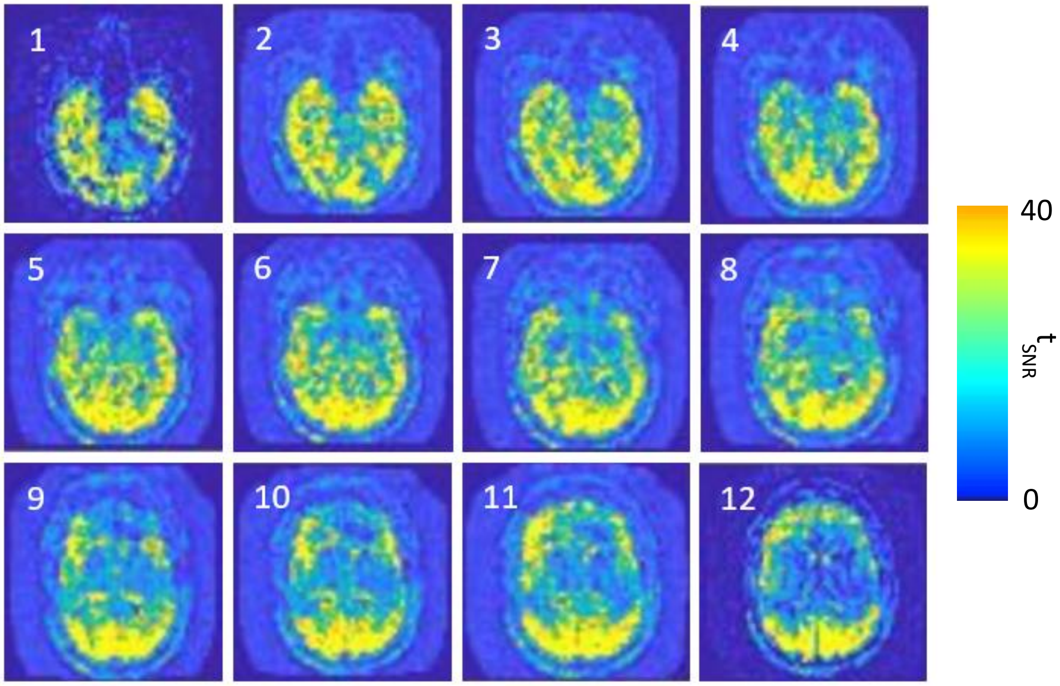

Supplementary Figure 3

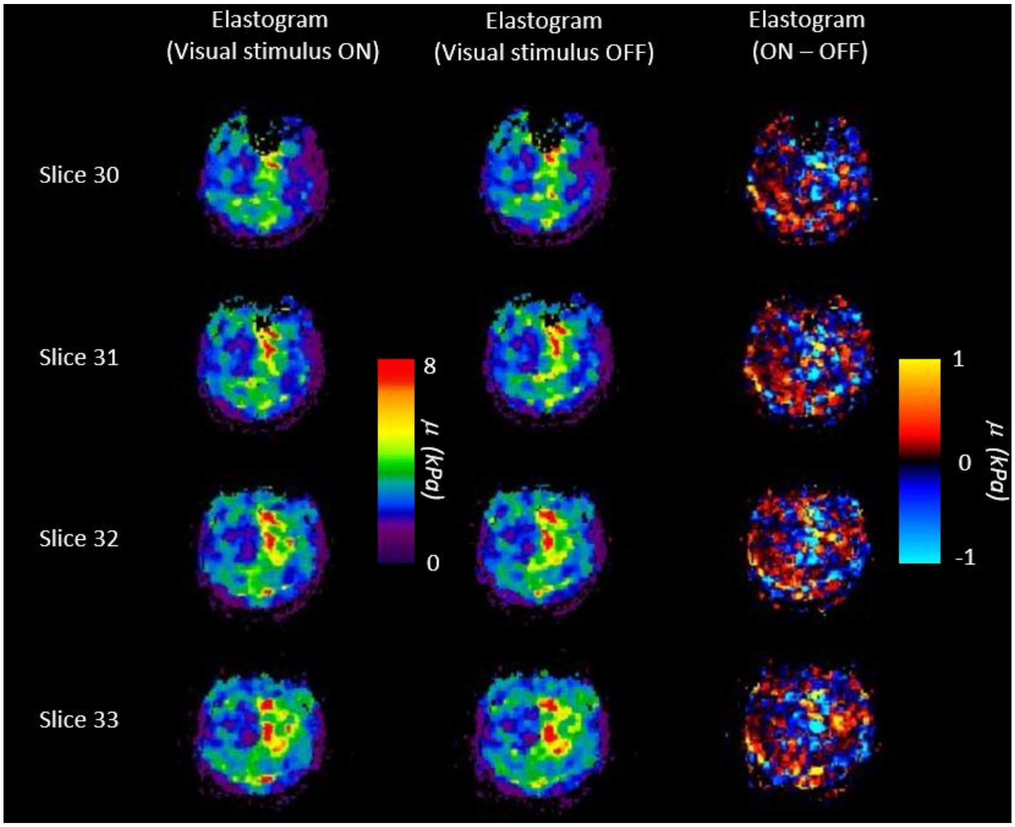

Supplement: Supplementary Material [file imag_a_00172-supp.pdf]
